# Watching a movie or listening to music is effective in managing perioperative anxiety and pain: a randomised controlled trial

**DOI:** 10.1007/s00167-023-07629-z

**Published:** 2023-10-28

**Authors:** Hafize Demirci, Sebastiaan L. van der Storm, Nathalie J. Huizing, Morgianne Fräser, Sjoerd A. S. Stufkens, Rover Krips, Gino M. M. J. Kerkhoffs, Esther Z. Barsom, Marlies P. Schijven

**Affiliations:** 1 Department of Surgery, Amsterdam UMC Location University of Amsterdam, Meibergdreef 9, Amsterdam, The Netherlands; 2 Amsterdam Gastroenterology and Metabolism, Amsterdam, The Netherlands; 3 Amsterdam Public Health, Digital Health, Amsterdam, The Netherlands; 4 Amsterdam UMC, Department of Orthopedic Surgery, University of Amsterdam, Amsterdam Movement Sciences, Amsterdam, The Netherlands; 5 Academic Center for Evidence-Based Sports Medicine (ACES), Amsterdam, The Netherlands; 6 Amsterdam Collaboration for Health and Safety in Sports (ACHSS), AMC/VUmc IOC Research Center, Amsterdam, The Netherlands; 7 Department of Orthopaedic Surgery, Flevoziekenhuis, Almere, The Netherlands

**Keywords:** Distraction therapy, Audiovisual distraction, Audio distraction, Orthopaedic surgical procedure, Local anaesthesia, Regional anaesthesia, Video glasses, Music

## Abstract

**Purpose:**

Despite the use of perioperative anxiolytics and pain medication, surgery can be a stressful and painful experience. Providing patients with distractions using video and/or audio tools in addition to medication may be helpful. To date, no studies have compared different distraction modalities in a same-day surgical setting in adults. This study aims to determine whether audio-visual distraction with video glasses (AVD) is more effective in reducing anxiety and pain compared to audio distraction (AD) in conscious patients undergoing orthopaedic surgery. It was hypothesised that AVD, being the more immersive modality, would be more effective than AD on the outcome parameters.

**Methods:**

Fifty patients undergoing orthopaedic surgery with local and/or regional anaesthesia in a clinical day-care setting were randomly assigned to receive either fixed-scenery AVD or patient-choice AD with music. Primary outcome was anxiety, as measured by the Dutch version of the Spielberger State–Trait Anxiety Inventory-6 (STAI-6) prior to and 15 min after the intervention. Secondary outcomes were pain (Numeric Rating Scale Pain [NRS-P]), systolic and diastolic blood pressure, heart rate and patient satisfaction.

**Results:**

Within each group, there was a significant reduction in anxiety (*p* = 0.028 for AVD, *p* < 0.001 for AD). In contrast to our hypothesis, listening to music without watching a video (AD group) reduced anxiety significantly more than experiencing full AVD (*p* = 0.018). The mean pain score did not change significantly within either user group, nor did pain scores differ between user groups.

**Conclusion:**

In conscious patients undergoing surgery, watching a movie (using video glasses and a headphone set) and listening to music (using only a headphone set) are able to significantly reduce anxiety. AVD, although believed to provide higher levels of distraction, did not prove to be superior to AD. The clinical relevance of this study highlights the potential benefits of AVD or AD modalities in improving the surgical experience for conscious patients. Further research is required to examine the influence of freedom of choice in content on the aforementioned outcomes. To estimate the true value of higher immersion levels, different distraction modalities (e.g. AVD versus virtual reality) featuring the exact same scenery or content need to be compared.

**Level of evidence:**

Level I.

**Supplementary Information:**

The online version contains supplementary material available at 10.1007/s00167-023-07629-z.

## Introduction

For many people, undergoing surgery is perceived as a stressful life event that often leads to anxiety in the perioperative period, which greatly exacerbated when a patient fears pain and a lack of control [5, 9]. To alleviate stress, shared decision-making and taking the time to explain risks and foresights are a key element of pre-operative counselling [48]. Nonetheless, patients’ uncertainties soften persist, with stress levels likely to peak when the actual moment of surgery arrives [41, 45].

Various studies have shown that high levels of perioperative anxiety increase post-operative pain; this then has a negative effect on subsequent healing and recovery and even increases susceptibility to infection [2, 8, 9, 26, 45]. Reducing pre-operative anxiety is, thus, considered the standard of care in anaesthesia. Anxiolytic drugs are often given before surgery to enhance the effect of anaesthetics [20]. Although effective, anxiolytics can lead to mild sedative side effects such as prolonged amnesia, drowsiness, and breathing difficulties, and they can therefore complicate same-day discharge [10, 43].As such, anxiolytics are mainly used in for in-patient surgeries and are avoided in short-stay or same-day (ambulant) surgeries [13]. However, same-day discharge is becoming increasingly feasible, especially for operations that require less sedation, due to developments such as the use of tranexamic acid, minimally invasive techniques, and preventive analgesia [7, 42].

Previous studies have identified alternative techniques that effectively reduce anxiety and the perception of pain, such as audio distraction (AD) and/or audio-visual distraction (AVD) [17, 23, 34, 46]. Listening to music in the immediate pre- and post-operative period has been shown to reduce anxiety levels [1, 13, 33, 54]. Recent studies indicate that AD, in particular music, has a positive effect on heart rate, respiratory rate, and blood pressure, inducing molecular responses that promote relaxation and reduce anxiety and pain in patients [12, 46].

Patients can receive AVD via variety of modalities, such as screens or monitors, video glasses with or without auditory headsets, and virtual- or augmented reality headsets. The use of AVD has been shown to be effective in reducing anxiety in numerous dental and paediatric care procedures and can reduce pain and the administration of sedative medication [15, 37, 38, 40]. It is often argued that AVD is more immersive than AD and, therefore may have a greater impact on stress and pain than AD, thereby improving patients’ overall experience when undergoing surgery in a conscious state [22, 24, 29, 44]. However, to our knowledge, no studies have examined the clinical benefits of comparing AVD and AD for adult patients in the same-day surgical setting. We hypothesise that AVD leads to more anxiety reduction compared to AD due to AVD’s more immersive nature.

This study addresses the potential benefits of AVD using the HappyMed® system (video glasses and a headset), versus AD using only headphones in relation to lowering anxiety and perceived pain in patients undergoing orthopaedic surgical procedures in a clinical day-care setting [53].

## Materials and methods

The study was approved by the regional medical ethics review committee of Amsterdam University Medical Center (UMC; METC 2016_009, Amsterdam, The Netherlands). The clinical research unit of the Amsterdam UMC (Amsterdam, The Netherlands) monitored the trial. This study was performed after informed consent was obtained.

### Trial design

A multicentre, randomised controlled trial was conducted between April 2016 and June 2022 in the Amsterdam UMC, Amsterdam Medical Center, and Flevo Hospital in the city of Almere, the Netherlands.

### Study participants

Patients eligible for study were scheduled for lower limb orthopaedic surgery under local and/or regional anaesthesia, were aged 18 years or older and had a surgical operation time of at least 30 min to be performed in a clinical day-care setting (same-day surgery). Patients were excluded when experiencing language barriers and when having a known history of hyper- or hypotension; substance abuse history; psychiatric history or physical and/or cognitive disabilities possibly affecting proper use of systems. Full eligibility criteria are provided in the Supplementary Appendix (Figure S1). Patients who received anaesthesia leading to loss of consciousness or who discontinued AVD or AD during sessions were considered lost to follow-up.

### Intervention

**Fig. 1:**
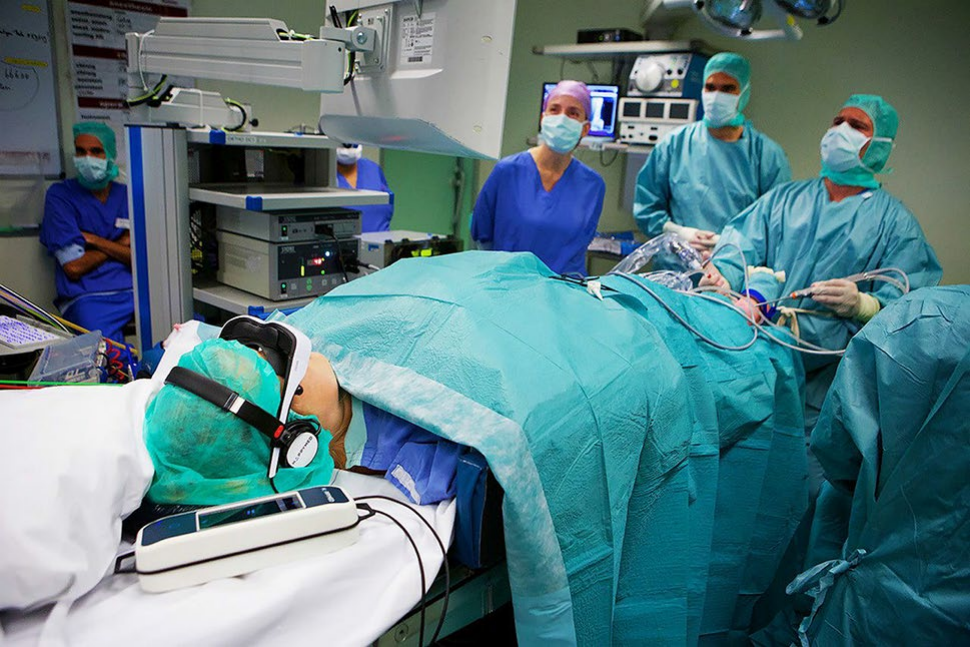
Patient undergoing surgery wearing the ‘HappyMed®’ AVD system in the operating room. Photo taken with consent of all involved

Participants were instructed not to take any anxiolytics within 24 h of surgery. Both intervention groups received the same standard of care for anaesthesia, surgical preparation, and medication. Randomisation took place in the operating room, minutes before surgery. During surgery, the AD group used over-the-ear adjustable headphones (Philips SBC HP200) to listen to their preferred type of music on Spotify or YouTube, with the volume controlled by the investigator. Patients in the AVD group did not have freedom of choice for preferred content. They all watched and listened to a Dutch wildlife documentary about the nature reserve ‘Oostvaardersplassen’ in the Netherlands, entitled ‘De Nieuwe Wildernis’ (the New Wilderness; a video having received several awards [51, 52]. This movie depicts animal life over four seasons and was presented using the HappyMed® system. This system includes a head-mounted display covering both eyes, is adjustable for individual eye conditions, and can be adapted to accommodate different pupil distances. The HappyMed® system offers surround-sound headphones to provide for auditory immersion, simulating a cinema experience that attempts to disconnect the user from their immediate surroundings (Fig. 1) [53]. The system was controlled by the investigator using a cable-connected media centre. Upon patients’ or healthcare providers’ request, the audio levels of both systems were adjusted by the investigator to allow for necessary communication. Unless specifically requested, the intervention was not interrupted. Any interruptions were documented. The intervention was initiated after time-out procedure and immediately before surgery started. Systems were activated before the first incision and deactivated after dressings were applied to the wounds, just before sign-out.

### Outcomes

The primary outcome of this study was the difference in experienced anxiety levels, as measured 15 min prior to the surgical intervention and 15 min after the sign-out timestamp of the procedure. Anxiety levels were measured using the Dutch version of the Spielberger State–Trait Anxiety Inventory-6 (STAI-6) [6]. The combined scores of the state- and trait-anxiety scale range from 20 to 80 points. A higher value reflects greater anxiety. In this study, a shortened Dutch version of the STAI-6 form was used, the ‘Zelf Beoordelings Vragenlijst’ (ZBV). This tool is considered valid and reliable to assess self-reported momentary anxiety in the Dutch patient setting [6, 50].

The secondary outcomes consisted of reported levels of pain, monitoring of vital signs, and reported patient satisfaction. The Numeric Rating Scale for Pain (NRS-P) was used to assess the level of pain immediately prior to surgery (baseline) and two hours after the surgical sign-out timestamp (0 = no pain,10 = worst pain possible). Vital signs included systolic blood pressure (SBP), diastolic blood pressure (DBP), and heart rate. Vital signs were measured preoperatively, intraoperatively (every five minutes) and post-operative (15 min and 2 h after surgery). Patient satisfaction was rated on a scale of 0–10 (0 = very unsatisfied, 10 = very satisfied). Additionally, patients were asked to rate their likelihood of recommending the intervention to others (0 = strongly disagree, 10 = strongly agree, Table 1).

**Table 1:** Time points and measurements

Measurements	T0	T1	T2	T3
STAI-6	X		X	
NRS-P	X			X
Systolic blood pressure (mmHg)	X	X	X	X
Diastolic blood pressure (mmHg)	X	X	X	X
Heart rate (beats per minute)	X	X	X	X
Recommendation grade			X	
Experience grade			X	

*T0 *pre-operative; *T1* intra-operative; *T2* 15 min post-operative; *T3* 2 h post-operative

### Randomisation and blinding

Patients were randomly assigned in a 1:1 ratio to either the AVD or AD intervention group using computer-generated randomisation with block sizes of 4 and 6. The coordinating research physician enrolled eligible patients with their permission, obtained informed consent, and conducted the randomisation. Blinding for intervention was not possible since the operative team and the patient were obviously aware of the assigned intervention after randomisation.

### Statistical analysis

Descriptive statistics were used to report variables, while Q–Q plots assessed normality. Mean and standard deviation (SD) were used for normally distributed continuous variables, and median and interquartile range (IQR) for non-normally distributed variables. Categorical variables were expressed as frequencies and percentages. Student’s *t*-test compared normally distributed continuous variables between two groups, while chi-square or Fisher’s exact test analysed categorical variables. The Mann–Whitney *U* test assessed non-normally distributed variables. Linear mixed models were used for variables with multiple paired measurements and linear regression was used to assess relationships between continuous variables and outcomes in both normal and non-normal distributions.

Variables with *p* < 0.100 in univariate analysis were considered possible predictors in linear regression, with ∆STAI-6 as the dependent variable. Multivariate linear regression included intervention, gender, age, duration of surgery, duration of intervention, mean SBP, mean pulse, previous surgery, VAS baseline and STAI-6 baseline as predictor variables. Non-significant variables were eliminated iteratively until only significant predictors remained (*p* < 0.050). No Bonferroni method was applied due to the limited number of variables. All statistical analyses were performed using the Statistical Package for the Social Sciences (SPSS) version 26.0 (IBM Corp., Armonk, New York).

The sample size calculation was based on the difference in the stress experience score as measured by the STAI-6 in the intervention and control groups. Based on past literature, we assumed that the use of AVD would be more immersive than AD and that during the surgery, AVD would reduce the level of anxiety with a mean difference of 6.00 compared to AD [57]. With a two-sided significance level of 5%, 80% power, a dropout rate of 10%, and an expected SD of 7.0, a total of 50 patients were needed.

## Results

**Fig. 2:**
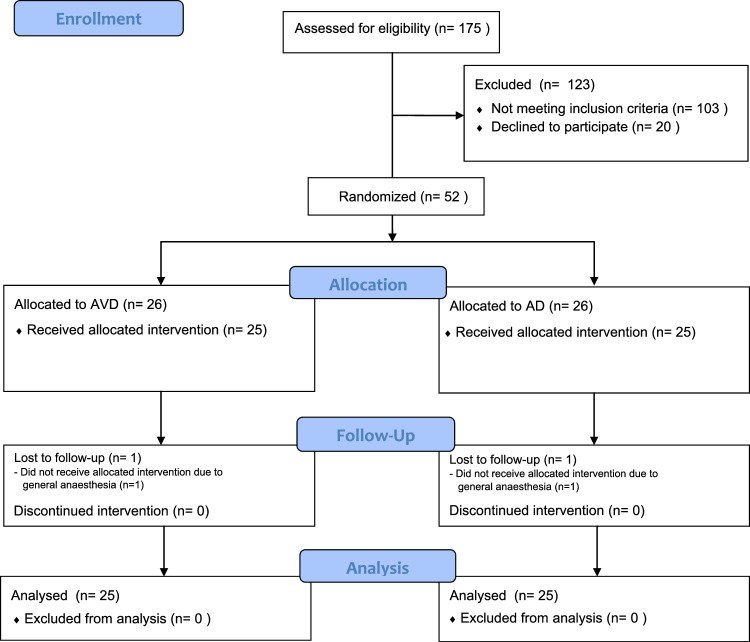
Flowchart of the study

In total, 175 participants were assessed for eligibility, of whom 52 were enrolled in the trial. Two patients were accidently oversampled. Finally, 50 participants received the assigned intervention, were included for analysis, and completed the study (AVD = 25 patients, AD = 25 patient; Fig. 2). Interruptions of the interventions occurred upon the request of the patient when needing interaction with the OR team or due to technical issues, which did not have a significant impact on the outcomes (non-significant [n.s.]). The mean age and difference in sex between patients in the AVD and AD groups appeared to differ significantly (*p* = 0.007, *p* = 0.023). No significant differences were retrieved considering clinical baseline characteristics, as displayed in Table 2.

**Table 2:** Demographic and clinical characteristics of participants

	AVD (*n* = 25)	AD (*n* = 25)	Total (*n* = 50)	*p*-Value
Sex, *n* (%)				0.023
Female	18 (72)	10 (40)	28 (56)	
Male	7 (28)	15(60)	22 (44)	
Age, mean ± SD	48.0 ± 12.6	37.2 ± 14.5	42.6 ± 14.5	0.007
Minimum	22	18	18	
Maximum	63	65	65	
Previous surgery, *n* (%)				
Yes	22 (88)	21 (84)	43 (86)	n.s.
DBP mmHg, median [IQR]	80.0 [71.5–87.0]	83.0 [75.5–89.5]	81.5 [72.8–87.3]	n.s.
SBP mmHg, median [IQR]	128.0 [109.5–141.5]	131.0 [117.0–147.0]	128.0 [114.8–143.5]	n.s.
HR bpm, mean ± SD	75.3 ± 14.5	70.1 ± 9.7	72.7 ± 12.5	n.s.
Duration of expected surgery^a^, median [IQR]	50 [41–70]	65 [41–80]	56 [41–72]	n.s.
Duration of intervention^a^, median [IQR]	31 [21.5–43.5]	35 [30–52.5]	33.5 [25.8–45.3]	n.s.
Intervention disturbed, *n* (%)				n.s.
No	22 (88)	16 (64)	38 (76)	n.s.
Yes, 1 time	3 (12)	7 (28)	10 (20)	n.s.
Yes, 3 times	0 (0)	2 (8)	2 (4)	n.s.
Time disturbed^a^, median [IQR]	0 [0.0–0.0]	0 [0.0–1.75]^b^	0 [0.0–0.0]^b^	n.s.
Grade experience, median [IQR]	9.0 [8.0–10.0]^b^	9.0 [8.0–9.0]	9.0 [8.0–10.0]	0.029
Grade recommendation, median [IQR]	9.0 [8.0–10.0]	9.0 [8.0–10.0]	9.0 [8.0–10.0]	n.s.

*AVD* audiovisual distraction; *AD* audio distraction; *BPM* beats per minute; *CI* confidence interval; *DBP* diastolic blood pressure; *HR* heart rate; *IQR* inter quartile range; *SBP* systolic blood pressure; *SD* standard deviation; *n.s.* non-significant

^a^In minutes, considered significant (*p* < 0.05)

^b^Missing *n* = 1

^c^Missing *n* = 2

### Primary outcome

Table 3 presents the comparison of anxiety levels, between the intervention groups (AVD and AD). The change in STAI-6 score appears to be significantly larger in the AD group than in the AVD group (*p* = 0.018). Table 4 includes a comparison of anxiety levels within the intervention groups (AVD and AD). Both the AVD and AD groups demonstrated a significant decrease in mean STAI-6 score within the group (MD -3.6, *p* = 0.028 for the AVD group, and MD- 8.6, *p* < 0.001 for the AD group).

**Table 3:** Comparison between subject measures for STAI-6

	AVD (*n* = 25)	AD (*n* = 25)	Total (*n* = 50)	Mean difference (95% Cl)	*p*-Value
STAI-6, mean ± SD					
Pre-operative	32.0 ± 8.5	34.6 ± 7.7	33.3 ± 8.1	2.5 (− 2.1 U 7.1)	n.s.
Female	32.6 ± 8.4	39.3 ± 6.6	35.0 ± 8.3	6.7 (0.3 U 13.0)	0.040
Male	30.6 ± 9.3	31.4 ± 6.7	31.1 ± 7.4	0.8 (− 6.4 ± 8.1)	n.s.
+ 15 min^a^	28.5 ± 8.7	25.9 ± 4.4	27.2 ± 6.9	− 2.5 (− 6.5 U 1.4)	n.s.
Female	28.0 ± 6.8	26.1 ± 4.1	27.3 ± 6.0	− 1.9 (− 6.8 U 3.0)	n.s.
Male	29.7 ± 12.9	25.8 ± 4.7	27.1 ± 8.1	− 3.9 (− 15.9 ± 8.1)	n.s.
∆ STAI-6	− 3.6 ± 7.6	− 8.6 ± 7.1	− 6.1 ± 7.7	− 5.1 (− 9.3 U − 0.9)	0.018
Female	− 4.6 ± 6.7	− 13.2 ± 7.0	− 7.7 ± 7.9	− 8.6 (− 14.1 U − 3.1)	0.004
Male	− 0.9 ± 9.6	− 5.6 ± 5.4	− 4.1 ± 7.2	− 4.7 (− 11.4 U 1.9)	n.s.

*AVD* audiovisual distraction; *AD* audio distraction; *SD* standard deviation; *CI* confidence interval; *n.s.* non-significant

Considered significant (*p* < 0.05)

^a^15 minutes post-operative

**Table 4:** Comparison within subjects measures for STAI-6

	STAI-6, mean ± SD		Mean difference (95% CI)	*p*-Value
	Pre-operative	+ 15 min^a^		
AVD	32.0 ± 8.5	28.5 ± 8.7	− 3.6 (− 6.7 U − 0.4)	0.028
Female	32.6 ± 8.4	28.0 ± 6.8	− 4.6 (− 8.0 U − 1.3)	0.010
Male	30.6 ± 9.3	29.7 ± 12.9	− 0.9 (− 9.8 U 8.0)	n.s.
AD	34.6 ± 7.7	25.9 ± 4.4	− 8.6 (− 11.6 U − 5.7)	< 0.001
Female	39.3 ± 6.6	26.1 ± 4.1	− 13.2 (− 18.2 U − 8.2)	< 0.001
Male	31.4 ± 6.7	25.8 ± 4.7	− 5.6 (− 8.6 U − 2.6)	0.001
Total	33.3 ± 8.1	27.2 ± 6.9	− 6.1 (− 8.3 U − 3.9)	< 0.001
Female	35.0 ± 8.3	27.3 ± 6.0	− 7.7 (− 10.7 U − 4.6)	< 0.001
Male	31.14 ± 7.4	27.05 ± 8.1	− 4.1 (− 7.3 U − 0.9)	0.014

*AVD* audiovisual distraction; *AD* audio distraction; *SD* standard deviation; *CI* confidence interval; *n.s.* non-significant

Considered significant (*p* < 0.05)

^a^15 minutes post-operative

### Secondary outcomes

**Fig. 3:**
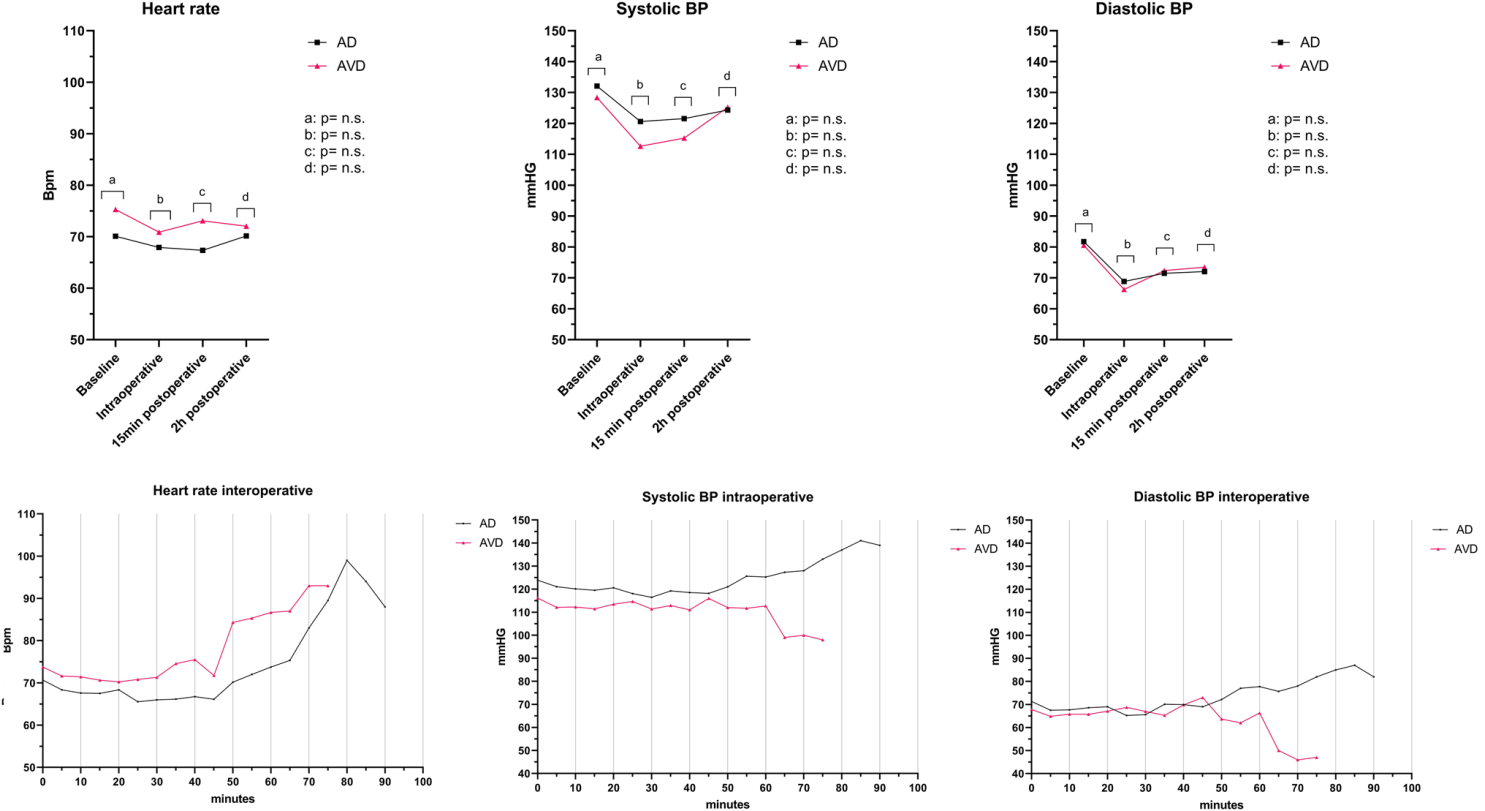
Results of the systolic blood pressure diastolic blood pressure, and pulse rate

No significant difference was observed in NRS-P preoperatively, NRS-P 2 h postoperatively, or ∆NRS-P between or within the intervention groups, respectively (Table 5). Figure 3 shows no significant differences between both groups regarding SBP, DBP, and HR. In the total population, three patients received anxiolytics (Temazepam), of which all three were in the AVD group (female *n* = 2, male *n* = 1).

**Table 5:** Comparison between and within subject measures for NRS-P

	AVD (*n* = 25)		*p*-value^c^	AD (*n* = 25)		*p*-value^c^	*p*-value^d^	Total (*n* = 50)		*p*-value^c^
	Pre	+ 2h^e^		Pre	+ 2h^e^			Pre	+ 2h^e^	
	∆ NRS-P			∆ NRS-P				∆ NRS-P		
NRS-P, median [IQR]	1.0 [0.0–3.0]^b^	0.0 [0.0–3.9]^a^	n.s.	1.5 [0.0–3.0]^a^	2.5 [0.0–4.0]^a^	n.s.	n.s.	1.0 [0.0–3.0]	2.0 [0.0–4.0]	n.s.
	0.0 [−2.0 to 2.0]			0.0 [−2.5 to 3.0]			n.s.	0.0 [−1.5 to 2.5]		

*AVD* audiovisual distraction; *AD* audio distraction; IQR interquartile range; *NRS-P* numeric rating scale for pain; *n.s.* non-significant

Non-parametric Mann–Whitney *U* test was performed, considered significant (*p* < 0.05)

^a^Missing *n* = 1

^b^Missing *n* = 2

^c^Within-group comparison

^d^Between-group comparison

^e^2 hours post-operative

### Predictors of outcome

Univariate analysis is provided in Table S2 in the Supplementary Appendix, variables with *p* < 0.100 can be interpreted as possible outcome predictors. A multiple linear regression was calculated to predict ∆STAI-6 based on their intervention, sex, and STAI-6 baseline (Table 6). After adjusting for sex and the STAI-6 baseline, the regression coefficient of the dependent variable ∆STAI-6 for the independent variable intervention was still significant (*p* = 0.009). Additionally, the overall model variation explained by the covariates when adjusted for overfitting was 44.2% (*R*^2^ = 0.442).

**Table 6:** Multivariate analysis, ∆STAI-6 as dependent variable

	Regression coefficient β	95% CI interval		*p*-value
		Lower	Upper	
Intervention^a^	5.080	0.898	9.262	0.018
Intervention^b,c^	4.889	1.303	8.475	0.009
Sex^d^	− 3.275	− 6.949	0.399	n.s
STAI-6 baseline	− 0.492	− 0.709	− 0.274	< 0.001

*∆STAI-6* STAI-6 anxiety score 15 min post-operative – STAI-6 anxiety score pre-operative; *n.s.* non-significant

^a^crude analyses, constant =  − 8.640

^b^Adjusted analyses, constant = 9.665. Adjusted R^2^ = 0.442

Intervention: ^c^AD = baseline, Sex: ^d^Male = baseline considered significant (*p* < 0.05)

## Discussion

The most important finding of this study highlights a noteworthy reduction in anxiety among patients in both the AVD and AD groups, with statistically significant results (*p* = 0.028, *p* < 0.001, respectively). This study is the first to compare the effects of AVD and AD on the levels of anxiety and pain in patients undergoing orthopaedic ambulant surgery.

### Main outcome

Our hypothesis that a higher level of immersion would lead to better distraction and, as a consequence, a greater reduction of anxiety and/or pain, proved not to be supported by our findings. On the contrary, the results revealed a significant decrease in anxiety among patients in the AD group compared to those in the AVD group (*p* = 0.018). Our findings indicate that female sex and a higher STAI-6 baseline may have an influence on outcomes. However, even after adjusting for these confounders, patients in the AD group still exhibit a significantly greater reduction in anxiety compared to their peers in the AVD group (*p* = 0.009).

Previous studies have established positive effects for both distraction methods to reduce anxiety and pain in clinical settings [14, 57]. Many studies using music interventions have demonstrated positive outcomes for perioperative anxiety and often use the STAI measurements [10, 11, 13, 37, 54–57]. There is limited literature available on the use of video glasses for distraction purposes, but what can be retrieved supports that these systems can effectively reduce anxiety and pain, as shown in a meta-analysis [48].

To date, only a few studies have compared music and video to distract patients during medical procedures and investigate these tools’ impact on anxiety and pain [21, 36]. However, this is the first study to compare AVD using glasses and AD using a headset in intraoperative patients in a same-day surgical setting. Gupta et al. found AVD to be most effective during cystoscopy, while Lee et al. found that AVD with video glasses reduced the need for sedation during endoscopic procedures (*p* < 0.001) [21, 36]. In addition, the use of video glasses has been shown to improve the overall patient experience [14]. While a step-by-step immersion to deepen the experience is believed to be most effective, video glasses with headphones are expected to close off the patient from their surroundings, thus being more effective than listening to music with headphones alone [3]. To our surprise, the results of this study indicated quite the opposite. This can partly be explained by a skewed distribution of sex between the intervention groups, sex randomisation, the STAI-6 baseline, the opportunity of freedom of choice, and the influence of a fixed type of content in the AVD group.

The AVD group had significantly more female patients compared to the music group. Studies indicate that females have a higher sensitivity to anxiety than males [32]. Surprisingly, female patients in the AVD group displayed a lower baseline STAI-6 score, with a mean difference of 6.7 points, compared to female patients in the music group (*p* = 0.040). Notwithstanding this initial disparity, it is important to note that the change in STAI-6 scores among females within the AVD group demonstrated statistical significance (*p* = 0.010), which could have influenced this study results. Nevertheless, these findings underscore the efficacy of the intervention in addressing anxiety levels within the study population while also highlighting potential sex differences in the response to the treatment.

Studies have assessed the relevance of familiarity and preference of type of content for relaxation [49]. Self-selected music appears to reduce anxiety and pain [8, 37]. Patients also indicate that they prefer choosing their own type of audio–visual distraction [16]. In this study, patients were able to choose their own type of music but not their own video. Although some patients found the movie ‘*De Nieuwe Wildernis*’ unentertaining, patients in the AVD group graded their overall experience significantly higher than patients in the AD group. Giving AVD patients control over their audio–visual choices is believed to increase the effectiveness of this treatment modality. However, it is noteworthy that, there was a significant reduction in patients’ anxiety even when patients could not choose their own movie.

Indeed, in medical virtual reality (VR) applications, having a choice of scenery has been shown to influence and alleviate anxiety and pain [4, 18]. In this study, we deliberately preferred video glasses as a distraction method over VR to avoid issues like unintended body movements and cybersickness [31, 35].

### Secondary outcomes

Silva et al. determined that during colonoscopy, listening to music reduced patients’ pain experience significantly more compared to video glasses [39]. However, this study did not observe a decrease in pain scores. This could be attributed to the initially low baseline pain scores in this study population (between 0 and 3). Using a more accurate pain measurement tool like the Visual Analogue Scale may enhance its significance [25]. Another important point is that the NRS-P was taken two hours postoperatively, so there is a good chance that the effects of the epidural anaesthetics had not fully worn off. No difference was observed in heart rate or blood pressure, though pas literature has reported that self-selected music can also have a positive effect on these [47, 57]. Video glasses distraction techniques have been found to have a positive effect on overall satisfaction with outpatient procedures, which is consistent with the results of this study [14, 23, 30]. The use of AVD therapy was graded significantly higher than AD therapy (*p* = 0.029).

Moreover, pre-operative anxiety and pain have the potential to prolong the post-operative recovery process, leading to an extended hospital stay and increased follow-up expenses [19, 27, 28]. Based on our results and current literature, we hypothesise that both AVD and AD can positively affect the post-operative recovery process, resulting in a shorter hospital stay and reduced costs.

There are certain aspects to consider in this study. Although the groups were randomly assigned, the difference in the male–female ratio between groups suggests that a post-hoc stratification by sex may have been necessary to further evaluate the effect of sex on outcome. However, the small sample size of this study precludes reliable adjustment for such effect modification. Although a power calculation was performed based on extant literature for the primary outcome, a larger population sample is likely to strengthen the significance of the results, leading to more consistency in studying the effects found in the literature and greater adjustment for more confounders. It is worth noting that we did not use a cut-off value for the level of anxiety during inclusion, so non-anxious patients were also included in the study. Furthermore, due to the replacement of the initial coordinating investigator, the COVID-19 pandemic, and the strict eligibility criteria, the trial took much longer than expected.

Next studies should focus on exploring the influence of different types of content and freedom of choice and comparing video glasses and VR glasses.

## Conclusion

Both AVD and AD therapy effectively reduce anxiety in patients undergoing orthopaedic surgery under local and/or regional anaesthesia. Based on our results, AD with content selection was more effective than AVD without content selection. It seems that HappyMed® video glasses and music therapy are both valid solutions for lowering anxiety in orthopaedic day-care patients; this may open up the discussion about modalities, giving more patients the opportunity to have local and/or regional anaesthesia instead of general anaesthesia for selected surgical procedures. Future studies should include a larger study population, investigate the impact of several types of content, improve sex randomisation for both groups, compare video glasses and VR glasses (including the effect of freedom of choice for content), and use an anxiety threshold as an inclusion criterion.

### Supplementary Information

Below is the link to the electronic supplementary material.

Supplementary file1 (DOCX 17 KB)

Supplementary file2 (DOCX 13 KB)

## Data Availability

Not applicable.
