# Supplementary material for: Watching a movie or listening to music is effective in managing perioperative anxiety and pain: a randomised controlled trial
Source: Knee Surg Sports Traumatol Arthrosc. 2023 Oct 28;31(12):6069–79. doi: 10.1007/s00167-023-07629-z (PMC10719121; doi:10.1007/s00167-023-07629-z)
Supplement: Supplementary file 1 — Supplementary file1 (DOCX 17 KB) [file 167_2023_7629_MOESM1_ESM.docx]

| **Table S2**. Univariate analyses, ∆STAI-6 as dependent variable | | | | |
| --- | --- | --- | --- | --- |
|  |  | **95% CI interval** | |  |
|  | **Regression coefficient β** | **Lower** | **Upper** | ***p*-value** |
| Intervention^1^ | 5.08 | 0.227 | 8.33 | .018* |
| Sex^2^ | -3.59 | -7.931 | 0.756 | .103* |
| Age | 0.005 | -0.149 | 0.159 | .949 |
| Duration of surgery from time out to sign out | 0 | -0.002 | 0.002 | .857 |
| Duration of intervention | -0.001 | -0.003 | 0.001 | .307 |
| Mean SBP^3^ to 15 min post-operative | -0.098 | -0.233 | 0.037 | .151 |
| Mean pulse to 15 min post operate | 0 | -0.199 | 0.199 | .998 |
| Previous surgery | 0.714 | -5.672 | 7.101 | .823 |
| VAS baseline | 0.616 | -0.562 | 1.794 | .298 |
| STAI-6 baseline | -0.588 | -0.804 | -0.371 | <.001* |
| ^1^AD=baseline  ^2^Male=baseline  ^3^Systolic Blood Pressure (SBP)  * were considered as possible predictors | | | | |

| **Table S1**. Univariate analyses, ∆STAI-6 as dependent variable | | | | |
| --- | --- | --- | --- | --- |
|  |  | **95% CI interval** | |  |
|  | **Regression coefficient β** | **Lower** | **Upper** | ***p*-value** |
| Intervention^1^ | 5.08 | 0.227 | 8.33 | .018 |
| Sex^2^ | -3.59 | -7.931 | 0.756 | .103 |
| Age | 0.005 | -0.149 | 0.159 | .949 |
| Duration of surgery from time out to sign out | 0 | -0.002 | 0.002 | .857 |
| Duration of intervention | -0.001 | -0.003 | 0.001 | .307 |
| Mean SBP^3^ to 15 min post-operative | -0.098 | -0.233 | 0.037 | .151 |
| Mean pulse to 15 min post operate | 0 | -0.199 | 0.199 | .998 |
| Previous surgery | 0.714 | -5.672 | 7.101 | .823 |
| VAS baseline | 0.616 | -0.562 | 1.794 | .298 |
| STAI-6 baseline | -0.588 | -0.804 | -0.371 | <.001 |
| Given anxiolytic | -0.603 | -9.937 | 8.732 | 0.897 |
| ^1^Music=baseline  ^2^Male=baseline  ^3^Systolic Blood Pressure (SBP) | | | | |
