# Supplementary material for: Watching a movie or listening to music is effective in managing perioperative anxiety and pain: a randomised controlled trial
Source: Knee Surg Sports Traumatol Arthrosc. 2023 Oct 28;31(12):6069–79. doi: 10.1007/s00167-023-07629-z (PMC10719121; doi:10.1007/s00167-023-07629-z)
Supplement: Supplementary file 2 — Supplementary file2 (DOCX 13 KB) [file 167_2023_7629_MOESM2_ESM.docx]

**Inclusion criteria:**

- Individuals scheduled for lower extremity orthopaedic surgery with an expected operative time of at least 30 minutes, under regional anaesthesia
- Aged 18 years or older

**Exclusion criteria:**

- Individuals with a known history of panic attacks, phobias, anxiety or borderline disorders as reported by patient in history taking or from patient files
- Individuals not being able to understand Dutch language at primary school level
- Individuals not being able to read or write Dutch
- Individuals with a known history of known hyper- or hypotensia
- Individuals with a history of loss of central or pheripheric field of vision on either eye
- Individuals with a history of either conductive, sensorineural or mixed hearing loss
- Individuals with refraction anomaly on either eye or both eyes, wearing glasses
- Individuals wearing hearing devices on either ear
- Individuals on any kind of anti-hypertensic mediation
- Individuals using any kind of prescribed or non-prescribed pain medication
- Individuals on any kind of anti-arrhythmic medication
- Individuals with a known history of alcohol, drug, and/or psychiatric problems
- Individuals who are unable to sign informed consent owing to mental disorder or formally stated to be incompetent to decide
- Individuals not willing or being able to sign informed consent for the proposed study
